# Genome-wide association study of leaf-related traits in tea plant in Guizhou based on genotyping-by-sequencing

**DOI:** 10.1186/s12870-023-04192-0

**Published:** 2023-04-12

**Authors:** Yanjun Chen, Suzhen Niu, Xinyue Deng, Qinfei Song, Limin He, Dingchen Bai, Yingqin He

**Affiliations:** 1 College of Tea Science / Tea Engineering Technology Research Center, Guizhou University, Guiyang, 550025 Guizhou Province People’s Republic of China; 2 Key Laboratory of Plant Resources Conservation and Germplasm Innovation in Mountainous Region, Ministry of Education, Institute of Agro-Bioengineering, Guizhou University, Guiyang, 550025 Guizhou Province People’s Republic of China; 3 School of Architecture, Guizhou University, Guiyang, 550025 Guizhou Province People’s Republic of China

**Keywords:** Tea plant, Leaf traits, Genome-wide association study (GWAS), Single nucleotide polymorphism (SNP), Candidate genes, Genotyping-by-sequencing (GBS)

## Abstract

**Background:**

Studying the genetic characteristics of tea plant (*Camellia* spp.) leaf traits is essential for improving yield and quality through breeding and selection. Guizhou Plateau, an important part of the original center of tea plants, has rich genetic resources. However, few studies have explored the associations between tea plant leaf traits and single nucleotide polymorphism (SNP) markers in Guizhou.

**Results:**

In this study, we used the genotyping-by-sequencing (GBS) method to identify 100,829 SNP markers from 338 accessions of tea germplasm in Guizhou Plateau, a region with rich genetic resources. We assessed population structure based on high-quality SNPs, constructed phylogenetic relationships, and performed genome-wide association studies (GWASs). Four inferred pure groups (G-I, G-II, G-III, and G-IV) and one inferred admixture group (G-V), were identified by a population structure analysis, and verified by principal component analyses and phylogenetic analyses. Through GWAS, we identified six candidate genes associated with four leaf traits, including mature leaf size, texture, color and shape. Specifically, two candidate genes, located on chromosomes 1 and 9, were significantly associated with mature leaf size, while two genes, located on chromosomes 8 and 11, were significantly associated with mature leaf texture. Additionally, two candidate genes, located on chromosomes 1 and 2 were identified as being associated with mature leaf color and mature leaf shape, respectively. We verified the expression level of two candidate genes was verified using reverse transcription quantitative polymerase chain reaction (RT-qPCR) and designed a derived cleaved amplified polymorphism (dCAPS) marker that co-segregated with mature leaf size, which could be used for marker-assisted selection (MAS) breeding in *Camellia sinensis*.

**Conclusions:**

In the present study, by using GWAS approaches with the 338 tea accessions population in Guizhou, we revealed a list of SNPs markers and candidate genes that were significantly associated with four leaf traits. This work provides theoretical and practical basis for the genetic breeding of related traits in tea plant leaves.

**Supplementary Information:**

The online version contains supplementary material available at 10.1186/s12870-023-04192-0.

## Background

Tea, made from the fresh and soft leaves of tea plants, has become a popular healthy beverage worldwide with high nutritional and medicinal value [1–3]. Tea plants are highly heterozygous and cross-pollinated. Conventional breeding approaches in tea depend on phenotypic selection and have been time-consuming because of tea’s relatively long juvenile stage. The mature leaf morphological traits of tea plants, such as leaf size, texture, shape, and color, are used regularly to determine genetic diversity among germplasm accessions, which play important roles in botanical classification, and tea yield and quality. The Guizhou Plateau, an important part of the original center of tea plants, has rich tea germplasms with strong genetic variation in morphological traits, especially in the leaf traits because of the three-dimensional ecological environment [4, 5]. Although some studies have addressed the leaf traits of tea germplasm in the Guizhou Plateau [6, 7], the genetic architecture and molecular bases of numerous leaf traits remain largely unknown. The functional genes of the leaf trait and the molecular markers in the functional genes have not yet been identified.

The tea plant is a major economic crop with buds and tender leaves as its product organ. The leaf traits are among the most important agronomic traits of the tea plant. The leaf size, texture, shape, and color play an important role in the yield, appearance, and quality of dried tea [8–10]. Breeding high-yield and suitable shape cultivars is a valuable goal for tea plant breeders. The variation of leaf traits is a complex mechanism arising from plants adapting to their environment. External factors (such as environment temperature, nutrient condition, and water supply) and internal factors (including cell division and cell expansion) contribute to the final leaf traits [11, 12]. Our comprehension of the genetic determinants for the mature leaf trait in tea plants remains primarily at the level of the genes found and their expression. Although some quantitative trait loci (QTLs) and single nucleotide polymorphisms (SNPs) associated with mature leaf size have been reported, the finding of genes and the development of functional molecular markers related to leaf traits remains insufficient for breeding [6, 7, 13, 14].

QTL mapping can be an effective tool for identifying genes underlying major leaf traits [15, 16]. Nevertheless, accurate QTL mapping depends on a large enough mapping population, which is not only a time-consuming process because of the long juvenile stage but the QTLs captured are limited because of the use of bi-parental hybridization in tea plants [2, 17]. Genome-wide association studies (GWASs), an association mapping method, have become a powerful tool for identifying multiple-related candidate genes that regulate major crop traits. GWASs are based on obtaining the genotype data of the research population and using high-density SNP markers with extensive genetic variation to predict the number of causative genes. It is based on the natural population to perform association analysis. The natural population has naturally hybridized for many generations and has sufficient recombination. This gives GWASs higher positioning accuracy [18]. GWASs have been successfully carried out in crops [19–21] and woody plants [22, 23]. Wang et al. [2] carried out GWAS of the timing of Spring Bud flush (TBF) on 151 tea plant germplasm resources, identified 26 SNPs and “Thymine. Thymine.” favorable allele variants, and designed a cleavage amplification polymorphism (dCAPS) marker associated with TBF. Yamashita et al. [24] conducted a GWAS on five metabolites related to tea quality from 150 tea plant materials and identified potential candidate genes controlling epicatechin (57 genes), epicatechin gallate (97 genes), epigallocatechin gallate (64 genes), total catechin (80 genes), and caffeine (83 genes). However, few studies have addressed the association analysis of leaf characteristics using the diverse tea germplasms and GBS sequencing method in Guizhou Province.

Molecular markers are essential for breeding major crops today, and many molecular marker techniques have been developed [25–27]. Among them, restriction fragment length polymorphism (RFLP), simple sequence repeat (SSR), amplified fragment length polymorphism (AFLP), and single nucleotide polymorphisms (SNPs) are the most effective marker systems for the determination of polymorphism, which are functionally responsible for specific traits or used to trace the evolutionary history of a species. SNPs are the most common source of genetic variation in eukaryotic species and have become an important marker for the genetic studies of plants. For example, 3.6 million SNPs were identified on 517 rice varieties using next-generation sequencing (NGS) with 19 × coverage. Taranto et al. [28] performed the genotyping-by-sequencing (GBS) for the genome-wide identification of SNPs in a collection of Capsicum spp. accessions. The results showed that 32,950 high-quality SNPs were identified on 222 C. annuum accession. Niu et al. [4] identified 79,016 high-quality SNPs at the genome level of 415 tea accessions using the GBS method.

The reduced cost and rapid progress in NGS and related bioinformatics resources promoted the large-scale discovery of SNPs in manly plants. GBS is the cost-effective NGS method to simultaneously discover and score segregating markers in populations of interest. GBS holds the potential to close the genotyping gap between references of broad interest and mapping/breeding populations of local or specific interest. The multiplexing of samples in GBS protocols keeps molecular biology costs low while the resultant next-generation sequencing data has immediate applications in many different research areas, ranging from gene discovery to genomic-assisted breeding [29].

In this study, 338 tea accessions were used from the Guizhou Plateau to identify high-quality SNPs for analyzing the population structure, linkage disequilibrium, and genetic diversity. The phenotype of the mature leaf trait and the genotype of 338 tea accessions were used to find the candidate genes and develop the molecular markers by GWAS. Our results provide useful information for future molecular marker-assisted breeding.

## Results

### Statistical analysis of phenotype variation

**Fig. 1:**
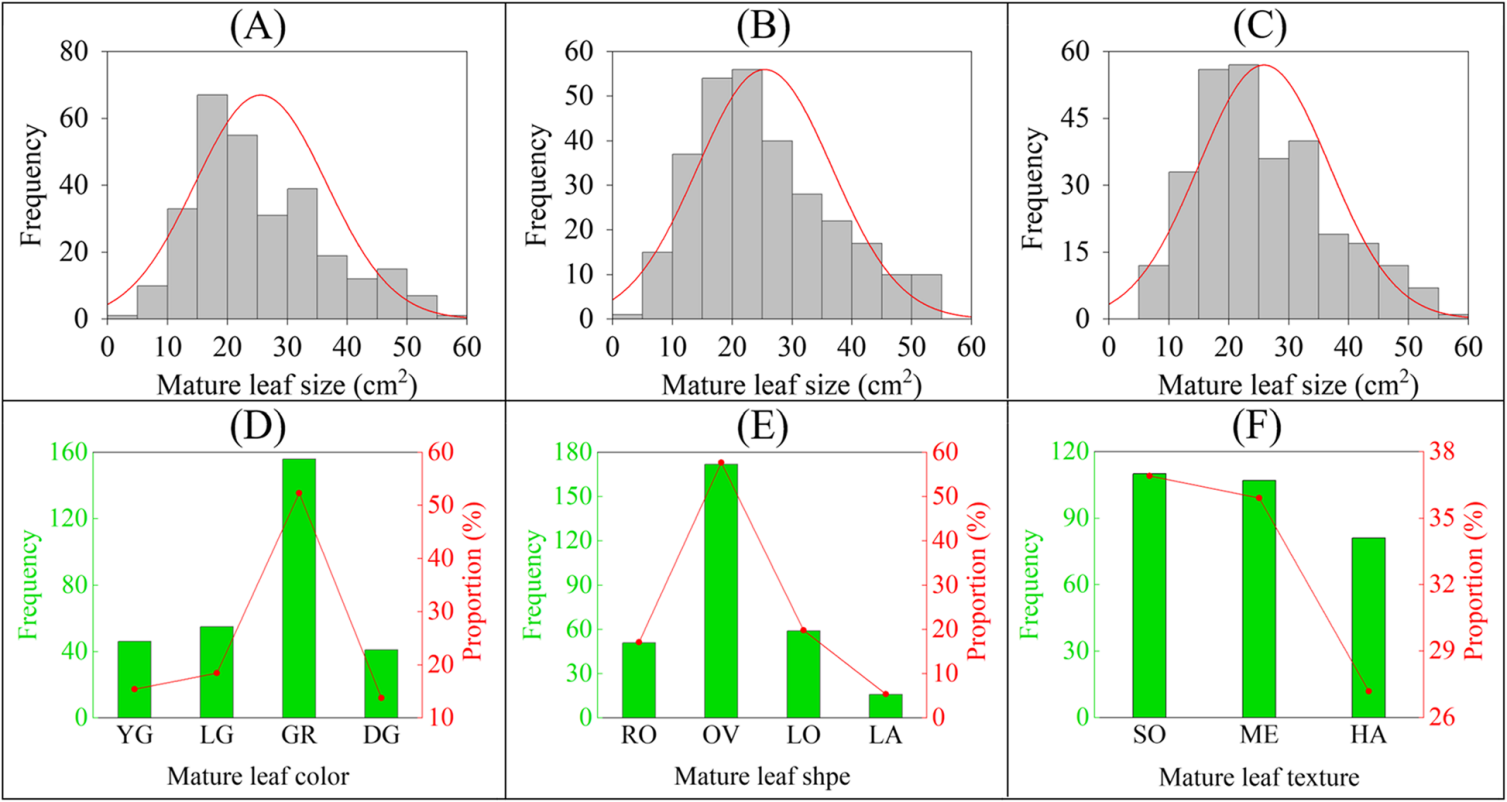
The frequency distribution of MLZ (in three years) and three qualitative traits (MLC, MLS, MLT), the frequency distribution of the three qualitative traits are mainly displayed by morphological characteristics. **A** Frequency distribution of MLZ in 2019. **B** Frequency distribution of MLZ in 2020. **C** Frequency distribution of MLZ in 2021. **D** Frequency distribution of MLC. Y.G.: yellow-green; L.G.: light green; G.R.: green; D.G.: dark green. **E** Frequency distribution of MLS. R.O.: round; O.V.: oval; L.O.: long oval; L.A.: lanceolate. **F** Frequency distribution of MLT. SO: Soft; ME: Medium; HA: Hard

To further understand the differences between different individuals for the same trait, we investigated four leaf traits of 338 tea plants in three years, including one quantitative trait (MLZ) and three qualitative traits (MLC, MLT, and MLS) [30]. We found that the four leaf traits of 338 tea plants displayed broad variation. For example, in the three years, the variation range of MLZ was 4.37 to 54.17 c m ^2^, 4.14 to 56.71 c m ^2^, and 6.14 to 55.34 c m ^2^, respectively, while the CV of MLZ were 48.5%, 46.6%, and 46.4%, respectively (Table 1). The 3-year frequency distribution of MLZ showed a normal distribution based on the best linear unbiased prediction (BLUP) (Fig. 1A-C). According to the descriptors for tea germplasm resources (NY/T 2943–2016), the three qualitative traits (MLC, MLT, and MLS) were mainly displayed by morphological characteristics in frequency distribution (Fig. 1D-F). The phenotypic characteristics of the three qualitative traits identified in this study were MLC (yellow green, light green, green and dark green), MLT (soft, medium and hard), and MLS (round, oval, long oval and lanceolate), which respectively account for 100%, 100% and 80% of the corresponding phenotypic characteristics in descriptors for tea germplasm resources (NY/T 2943–2016)(Table 2). These results suggested a broad diversity of the four leaf traits in 338 tea plants population.

**Table 1:** Statistical analysis of mature leaf size (MLZ) in the three years

Year	Min (c m ^2^)	Max (c m ^2^)	Mean ± SD (c m ^2^)	CV (%)
2019	4.37	54.17	23.16 ± 11.24	48.50
2020	4.14	56.71	23.51 ± 10.95	46.60
2021	6.14	55.34	23.41 ± 10.87	46.40

**Table 2:** Phenotypic identification statistical analysis of three qualitative traits

Traits	K		K’		PCP%	
Mature leaf color	4	11	4	12	100	91.67
Mature leaf texture	3		3		100	
Mature leaf shape	4		5		80	

*K* Phenotypic types identified, *K’* Phenotype types in NY/T 2943–2016, *PCP% = K/K’* The proportion of phenotypic types determined in this study which in descriptors for tea germplasm resources (NY/T 2943–2016)

The effects of genotype (G) and environment (E) on the MLZ of 338 accessions in the three years were studied by ANOVA. The difference in MLZ among 338 genotypes and within three years was significant (*p* < 0.05), indicating that the environment extensively influenced MLZ. The h_B_^2^ of MLZ was evaluated as 97.68%, and the stable inheritance was higher than 90% (Table 3).

**Table 3:** Variance analysis and broad-sense heritability (h_B_^2^) for mature leaf size (MLZ) of the 338 tea accessions

Source of variation	DF	ANOVA SS	Mean Square	F value	h_B_^2^
genotype (G)	287	104,095.08	356.70	127.14**	97.68%
environment (E)	2	25.71	13.05	4.65**	
error	574	1610.42	2.81		

^**^Represent *p* < 0.05

### Sequencing and variant discovery

**Fig. 2:**
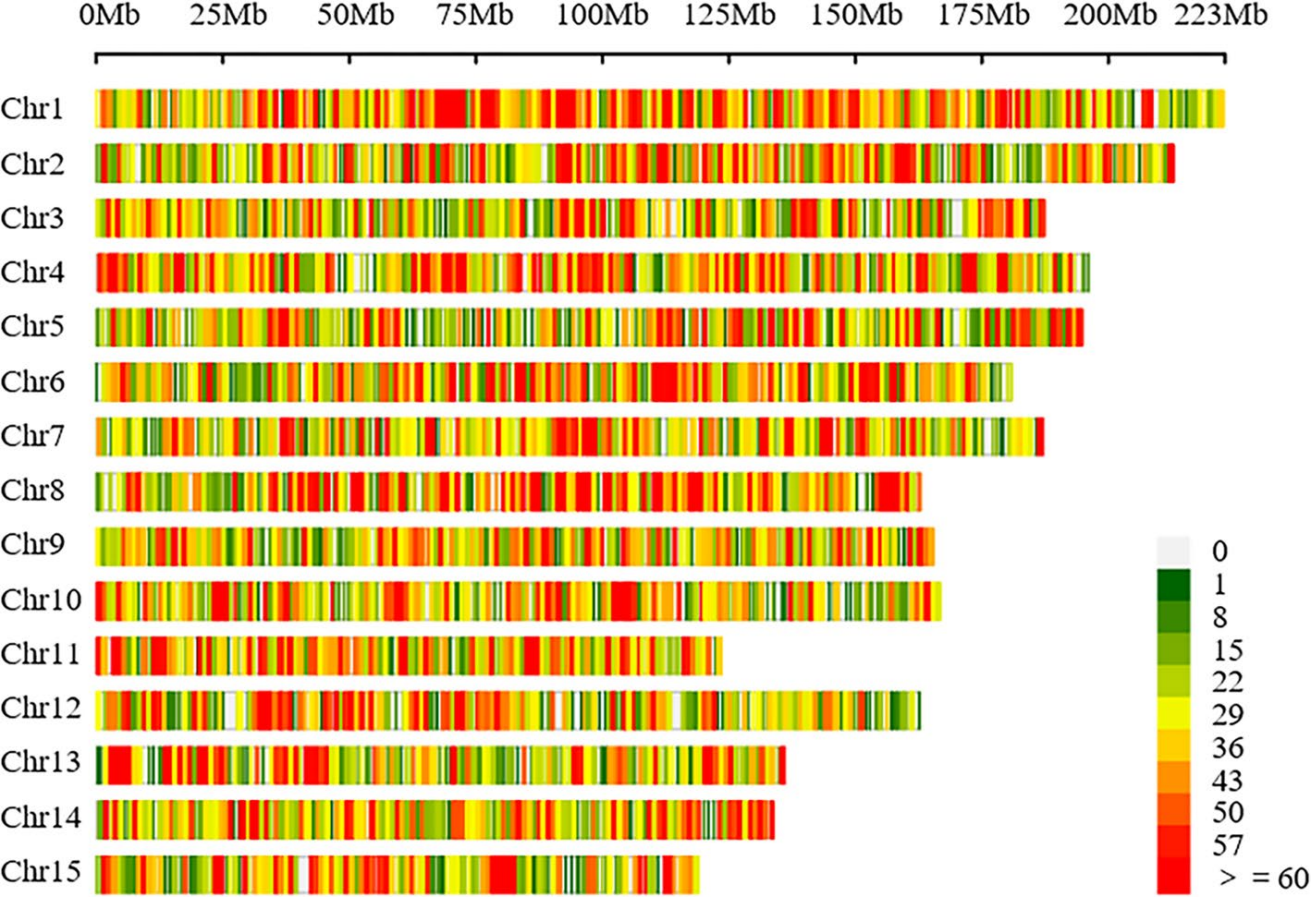
Distribution of single nucleotide polymorphisms (SNPs) on 15 chromosomes of the tea plant. The horizontal axis shows the chromosome length

GBS was performed on 338 tea accessions using Illumina HiSeq X ten. After the filtering step, 217 Gb clean data were generated with an average of 0.64 Gb per accession, and an average coverage depth was about 10 × (Table S1). Moreover, all clean reads were mapped on the released genome of the C. sinensis var. sinensis to identify the high-quality SNPs. In this study, a total of 29,393,327 SNPs were obtained from the 338 tea accessions, and 100,829 high-quality SNPs with a minor allele frequency (MAF) > 0.05 and missing data rate < 20% were conserved for subsequent analysis (Tables S2 and S3). Moreover, the distribution of high-quality SNPs was further investigated, and the results showed that 100,829 high-quality SNPs were roughly evenly distributed in 15 chromosomes of the tea genome (Fig. 2, Table S4). Chromosome 1 contained the largest number of high-quality SNPs (9140 SNPs), followed by chromosome 4 (7613 SNPs), and chromosome 15 had the smallest number of high-quality SNPs (4140 SNPs) (Table 4). The average number of high-quality SNPs on each chromosome was 6150. The lowest (32 SNPs/Mb) and highest (42 SNPs/Mb) SNP densities were detected on chromosomes 5 and chromosomes 8, respectively (Table 4).

**Table 4:** SNPs density of chromosome based on GBS

Chromosome	Length(bp)	SNP	Density(kb)
1	222,690,619	9140	41.04
2	212,893,553	7355	34.55
3	187,305,764	6286	33.56
4	196,128,943	7613	38.82
5	194,778,845	6165	31.65
6	180,870,292	6642	36.72
7	187,027,217	6354	33.97
8	162,894,436	6889	42.29
9	165,397,305	5645	34.13
10	166,753,223	5624	33.73
11	123,582,105	4771	38.61
12	162,686,597	5563	34.19
13	136,080,369	5054	37.14
14	133,792,454	5007	37.42
15	119,038,877	4140	34.78
Contig	2,540,239,288	8581	38.70
Total	3,021,230,785	100,829	38.24

### Genetic diversity estimation

Nucleotide diversity (*Pi*), minor allele frequency (*MAF*), observed heterozygosity (*Ho*), expected heterozygosity (*He*), and inbreeding coefficient (*Fis*) were used as indicators of genetic diversity. The *Pi*, *MAF, Ho*, *He* and *Fis* of 338 tea accession populations were 0.2268, 0.1448, 0.0721 0.2264 and 0.6936, respectively (Table 5). We compared the genetic diversity of five inferred populations of 338 Guizhou tea accession. *Pi, Ho, He* and *MAF* of G-V tea population were significantly higher than those of other tea populations, while *Pi*, *Ho*, *He* and *MAF* of G-III tea population were lower than those of other tea populations. *Fis* was higher for the tea population in G-III was higher than that of other tea populations (Table 5).

**Table 5:** Genetic diversity parameters of 338 tea accessions in Guizhou Plateau

Group	Number	Tajima's D	Pi	Ho	He	MAF	Fis
G-I	34	0.8588	0.1508c	0.0647c	0.1479c	0.1090c	0.5984a
G-II	24	0.2908	0.1402d	0.0596d	0.1340d	0.0932d	0.6287a
G-III	6	0.4549	0.1373e	0.0565e	0.1257e	0.0923d	0.6553a
G-IV	113	0.5016	0.1754b	0.0671b	0.1745b	0.1158b	0.5580a
G-V	161	0.8812	0.2254a	0.0814a	0.2245a	0.1448a	0.4724a
All	338	1.2618	0.2268	0.0721	0.2264	0.1448	0.6936

*Pi* nucleotide diversity, *Ho* observed heterozygosity, *He* expected heterozygosity, *MAF* minor allele frequency, *Fis* inbreeding coefficient; In the same type and line, the different letters indicate a significant difference in *p* = *0.05* levels by the T-test

Previous studies showed that when a positive Tajima's D value was determined for a population, the population was in a population bottleneck and/or balancing selection [31, 32]. The positive Tajima’s D values of these five tea populations here suggest that they all underwent population bottlenecks and/or balancing selection (Table 5).

### Population structure, PCA, and Phylogenetic analysis

**Fig. 3:**
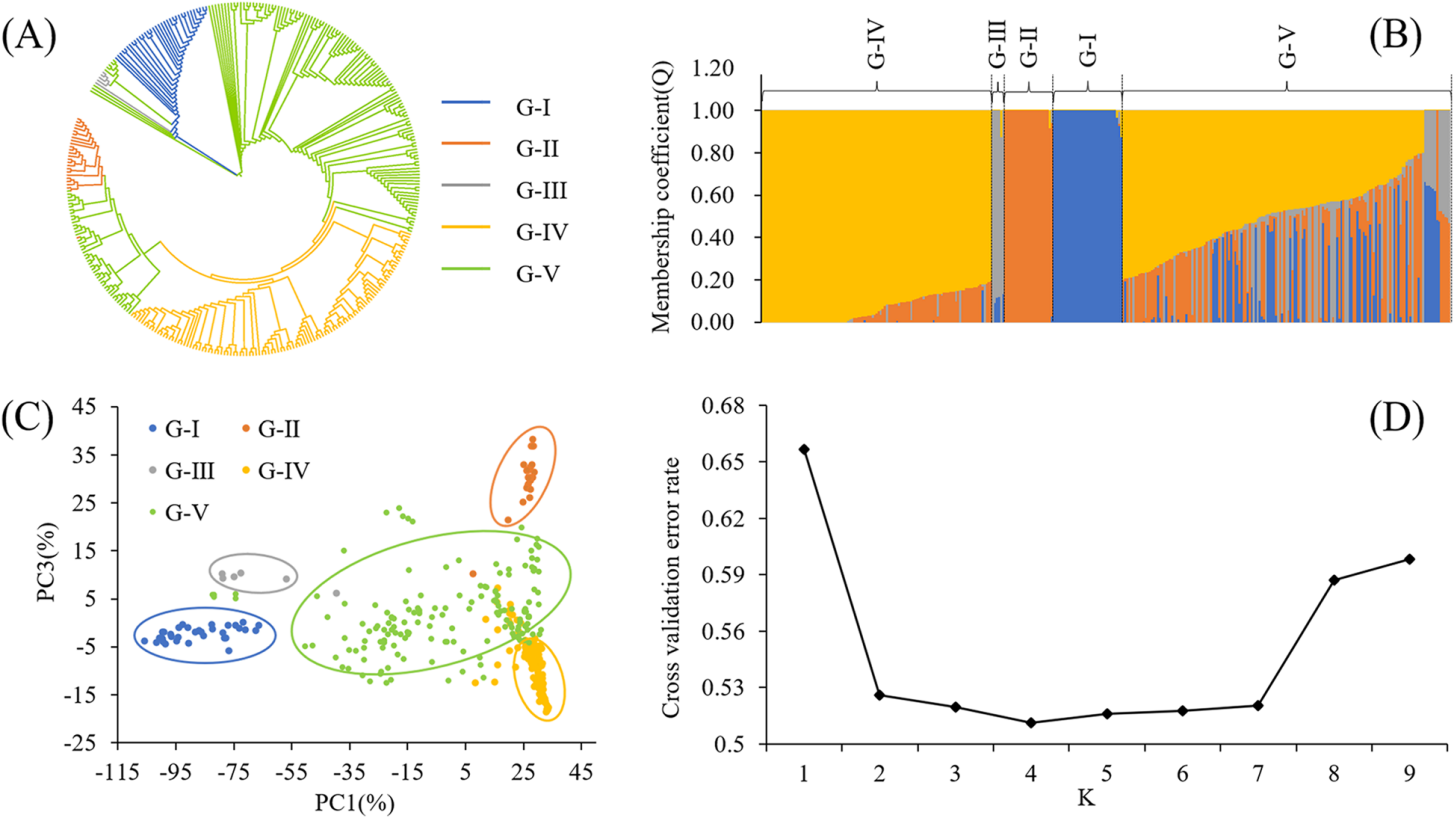
Population structure analysis, principal component analysis (PCA), and phylogenetic tree establishment of the 338 tea accessions. **A** The neighbor-joining tree was established by 100,829 high-quality SNPs. **B** Population structure separates the accessions into four subgroups (K = 4). **C** PCA of 338 tea accessions. And first and third components of the PCA analyses were shown; each dot represents an individual. **D** When K = 4, the CV error value was the smallest (0.51127)

To further explore the relationship of 338 tea plant populations, a total of 100,829 high-quality SNPs were used to perform the population structure analysis, PCA, and phylogenetic analysis. Dynamic changes in population structure were further evaluated under different K values (K = 2–9) (Figure. S1). Analysis of cross-validation error (CV error) under different K values revealed that the CV error was the smallest when K was equal to 4 (Fig. 3D). The membership coefficient 0.8 was used to distinguish pure ancestral and admixture groups. Accessions with the membership coefficient > 0.80 were assigned to the corresponding ancestral groups. Those with the membership coefficient < 0.80 were assigned to the admixture group (Fig. 3B). The 338 tea plants populations were further classified into five groups, four ancestral groups and one admixture group. The first ancestral group (‘*C. tachangensis* group’ or ‘G-I’) contained 34 *Camellia tachangensis* accessions (Tables S5 and S6). The second ancestral group (referred to as ‘Modern landraces group’ or ‘G-II’ from now on) contained 24 accessions, including 23 *C. sinensis* accessions mainly from the modern landraces (82.6%) and one *C. tachangensis* plant (Tables S5 and S6). The third ancestral group (referred to as ‘Transitional group’ or ‘G-III’ from now on) contained three *C. remotiserrata* plants and three *C. tachangensis* plants (Tables S5 and S6). The fourthancestral group (referred to as ‘Ancient landrace group or ‘G-IV’ from now on) contained 113 tea accessions, including 108 *C. sinensis* plants (91 tea accessions from the ancient landrace and 17 accessions from the modern landrace) and five *C. remotiserrata* plants (Tables S5 and S6). The admixture group (G-V) had 161 tea accessions, including 44 wild *C. remotiserrata* plants, 50 wild *C. tachangensis* plants, 63 cultivated *C. sinensis* plants, and four uncertain wild species (Tables S5 and S6).

To explore the cluster relationships and verify the stability of the potential population structure, principal component analysis (PCA) and Neighbor-Joining tree (NJ tree) was carried out on 100,829 SNPs of 338 tea accessions. According to the topology of the phylogenetic tree, the 338 tea accessions were mainly clustered into five groups: Group I (G-I), Group II (G-II), Group III (G-III), Group IV (G-IV), and Group V (G-V) (Fig. 3A). Among them, G-I contained the tea plants from Bijie, Zunyi, Qiannan Buyi and Miao, and Southwest Guizhou Autonomous Prefectures; G-II contained tea plants from Bijie, Guiyang, Liupanshui, Tongren, and Zunyi, Qiannan Buyi and Miao Autonomous Prefecture; G-III contained the members of tea accessions all from Zunyi City; G-IV contained tea accessions from Aushun, Bijie, Guiyang, Liupanshui, Tongren, Zunyi, Qiandongnan Miao and Dong, Qiannan Buyi and Miao, and Southwest Guizhou Autonomous Prefectures; G-V contained tea accessions from Guiyang, Tongren, Bijie, Zunyi, Qiandongnan Miao and Dong, and Southwest Guizhou Autonomous Prefectures. PCA disclosed five groups corresponding to G-I, G-II, G-III, G-IV and G-V (Fig. 3C). Therefore, the accuracy of the population structure was mutually verified.

### Linkage disequilibrium analysis

**Fig. 4:**
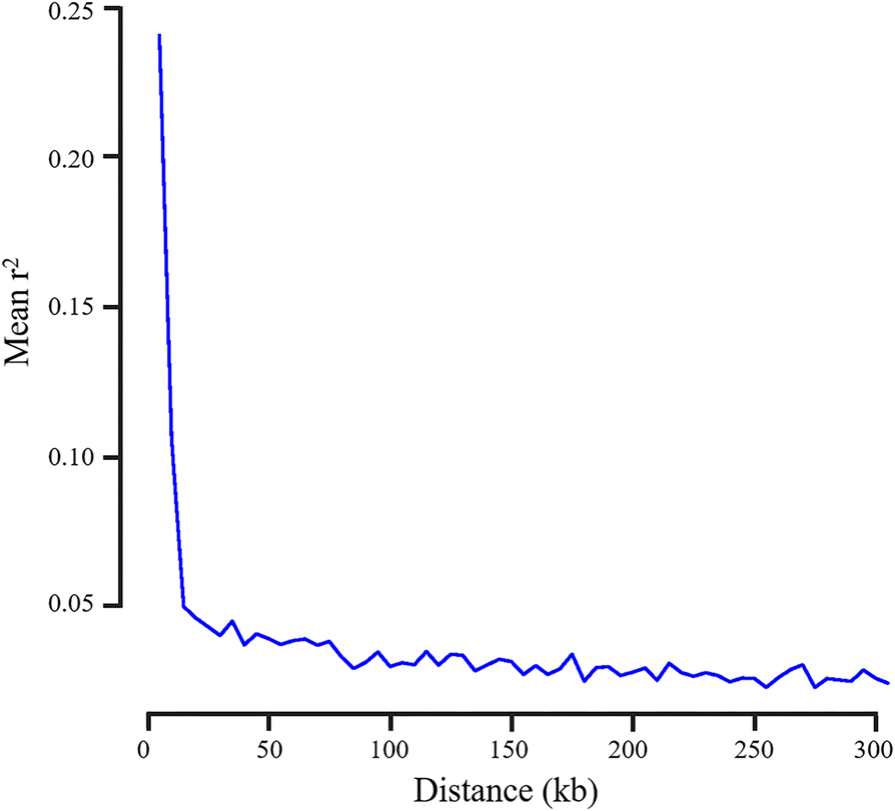
L.D. decay of 338 tea accessions

Linkage disequilibrium data can be used to detect associations between common variants and important traits. Linkage disequilibrium (LD, indicated by r^2^) analysis indicated that the 338 tea plants’ genomes had a relatively short r^2^ distance (~ 7 kb) and rapid r^2^ decay (Fig. 4). The r^2^ decreased to half its maximum value, at 7 kb. Therefore, we consider that 7 k b can be used as a search range of late candidate genes. As the average marker density was 30.7 kb (3.1 g/100,829) per SNP, we conclude that these selected SNPs were sufficiently dense to perform genome-wide association study.

### Genome-wide association study and candidate genes prediction

**Fig. 5:**
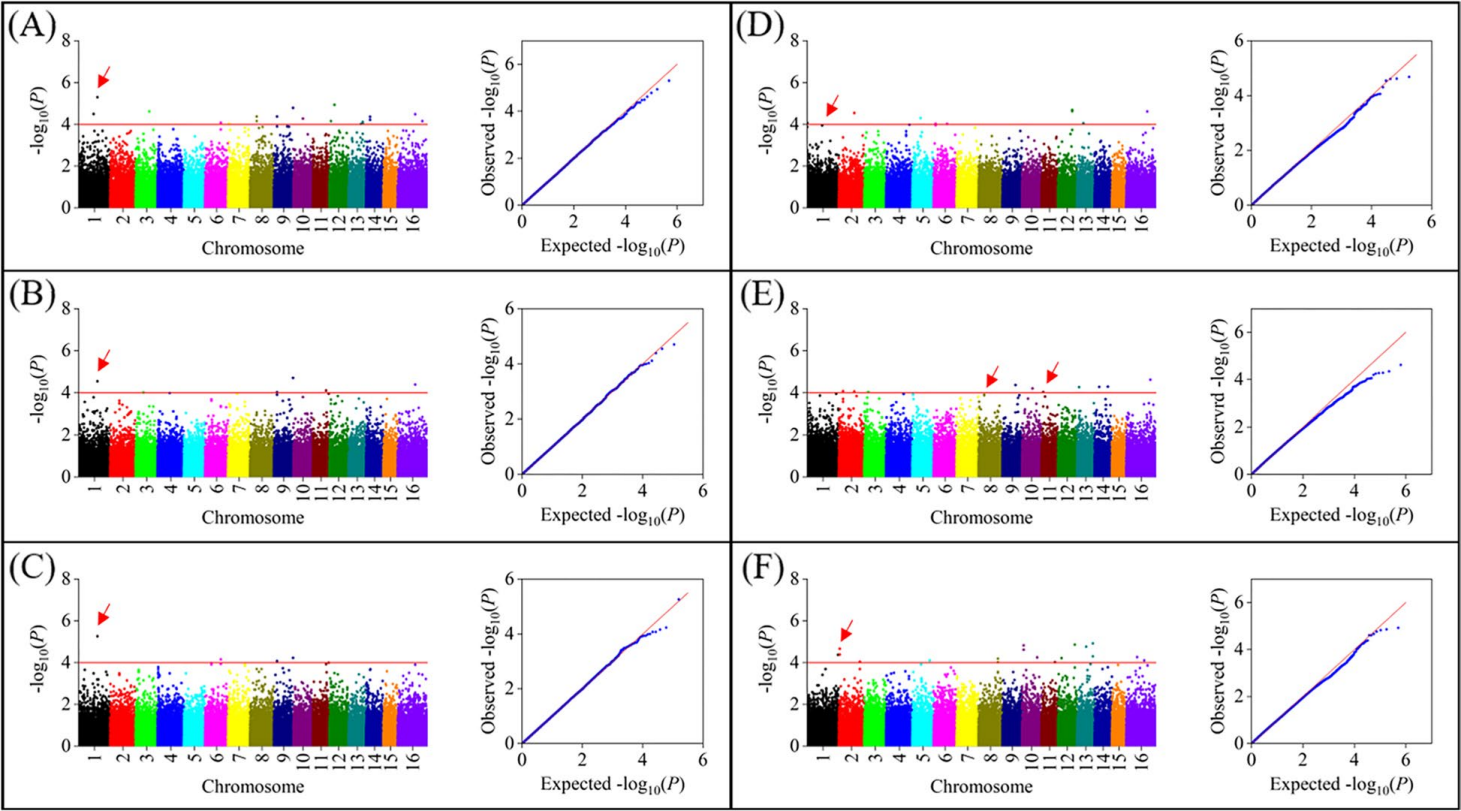
Manhattan plots and Q-Q plots of the 3-years (2019, 2020, 2021) cMLM-P + K model for MLZ traits of the tea plant. **A** MLZ-cMLM-P + K-2019; **B** MLZ-cMLM-P + K-2020; **C** MLZ-cMLM-P + K-2021; The red dashed horizontal line indicates the significance threshold (-log_10_^(P)^ is about equal to 4.0); The red arrows represent SNPS that have been jointly identified for MLZ traits in three years; “16” represents Contig (A continuous long DNA sequence overlapping fragment formed by joining short DNA fragments in the process of genome sequencing.) in Manhattan plots. Manhattan plots and Q-Q plots of the 1-year optimal model for three qualitative traits (MLC, MLS, MLT). **D** MLC-GLM-Q; **E** MLS-cMLM-P + K; (F) MLT-GLM-P; The red dashed horizontal line indicates the significance threshold (-log_10_^(P)^ is about equal to 4.0); The red arrows represent the ultimately significant SNPS associated with the three traits; “16” represents Contig (A continuous long DNA sequence overlapping fragment formed by joining short DNA fragments in the process of genome sequencing.) in Manhattan plots

Plant domestication conducted over several millennia had modified specific plant traits, especially leaf traits. To further identify the molecular mechanism involved in regulating four leaf traits, six linear regression models (GLM-Q, GLM-P, MLM-Q + K, MLM-P + K, cMLM-Q + K, and cMLM-P + K) were used to perform the GWAS using TASSEL5.0 software (Figure. S2). Comparing the Q-Q plots of the output of the six GWAS models, the best model fitting the curve of the expected value to the observed value was entered into the later analysis as the optimal model for each trait. Our result showed that cMLM-P + K, GLM-Q, GLM-P, and cMLM-P + K models were suitable for GWAS analysis for MLZ, MLC, MLT, and MLS, respectively (Fig. 5). A total of 59 high-quality SNPs were significantly associated with four leaf traits (− log_10_^(P)^ ≥ 4.0) (Tables 6 and 7). Among them, 9, 18, and 10 high-quality SNPs were significantly associated with MLC, MLT, and MLS, respectively (Table 6). And 22 high-quality SNPs were significantly associated with MLZ in three years (Table 7). Only one SNP (P-1076408) of nine high-quality SNPs was discovered in the coding region for MLC. Functional annotation of the coding sequence where the SNP is located revealed that the TEA027527.1 gene, encoding RAB geranylgeranyl transferase type 2 subunit β1, was involved in the biosynthesis of carotenoids and chlorophyll based on gene function annotation and KEGG analysis (Table 8). For MLT, ten SNPs of 18 high-quality SNPs were located in the coding region. Functional annotation of the coding sequence where the ten SNPs were located revealed that three SNPs (P-129108645, P-129108677, and P-101072100) associated with MLT were distributed in coding sequences of TEA021901.1 gene contained two SNPs (P-129108645 and P-129108677) and the TEA002469.1 gene (P-101072100), respectively. And they encoded Beta-glucosidase 12 based on gene function annotation of the TPIA and NCBI databases (Table 8). For MLZ, 22 high-quality SNPs were identified in the three years. Nine SNPs (P-122201532, P-116917717, P-124718592, P-163812618, P-100806529, P-11244003, P-92754559, P-110363923, and P-110363973) of 22 high-quality SNPs were distributed in the coding region. Functional annotation of the coding sequence where the nine SNPs were located revealed that two SNPs (P-122201532, P-163812618) were present in the coding sequences of the TEA005350.1 and TEA029641.1 genes, respectively. These two genes encode the TONSOKU protein and Pyrophosphate fructose 6-phosphate 1-phosphate transferase subunit α based on gene function annotation of TPIA and NCBI database (Table 8). For MLS, five SNPs (P-3090796, P-130091909, P-110799549, P-12773211, and P-107071402) of ten high-quality SNPs were discovered in the coding region. Functional annotation of the coding sequence where the five SNPs were located revealed that only one SNP (P-130091909) was distributed in the coding sequence of the TEA026128.1 gene, which was associated with MLS (Table 8).

**Table 6:** The optimal model of three qualitative traits of 338 tea accessions was used for SNPs analysis

Traits	Model	SNPs	Chromosome	Position	-Log_10_^(p)^	Allele	R^2^(%)
MLC	GLM(Q)	S1-P1076408	1	1,076,408	4.063	T/G	6.62
		S2-P128618813	2	128,618,813	4.543	G/A	7.43
		S5-P87525173	5	87,525,173	4.302	C/T	7.49
		S6-P112505345	6	112,505,345	4.018	A/G	7.65
		S6-P18460797	6	18,460,797	4.026	A/G	6.59
		S12-P108970717	12	108,970,717	4.621	A/G	8.56
		S12-P108970772	12	108,970,772	4.682	T/C	8.74
		S13-P38603004	13	38,603,004	4.056	G/A	7.31
		SCONTIG614-P349959	CONTIG614	349,959	4.610	G/A	7.71
MLT	GLM(P)	S1-P211517178	1	211,517,178	4.365	G/A	7.90
		S2-P11825464	2	11,825,464	4.655	G/A	8.36
		S2-P11825496	2	11,825,496	4.381	G/T	7.84
		S2-P171922400	2	171,922,400	4.046	G/A	7.66
		S5-P162371923	5	162,371,923	4.103	C/T	5.66
		S8-P129108645	8	129,108,645	4.020	A/G	6.53
		S8-P129108677	8	129,108,677	4.185	G/A	6.94
		S10-P119785042	10	119,785,042	4.252	G/A	7.53
		S10-P13082621	10	13,082,621	4.825	G/A	8.86
		S10-P13082689	10	13,082,689	4.609	C/A	8.36
		S11-P101072100	11	101,072,100	4.014	G/A	7.29
		S12-P32519734	12	32,519,734	4.214	A/G	7.29
		S12-P136348822	12	136,348,822	4.861	A/G	8.93
		S13-P55838677	13	55,838,677	4.758	T/C	7.92
		S13-P122895304	13	122,895,304	4.921	A/C	8.46
		S13-P122895559	13	122,895,559	4.302	A/G	7.30
		SCONTIG18-P388182	CONTIG18	388,182	4.263	A/G	6.98
		SCONTIG446-P62289	CONTIG446	62,289	4.108	A/T	7.38
MLS	cMLM(P + K)	S2-P40432827	2	40,432,827	4.062	G/T	7.21
		S2-P130091909	2	130,091,909	4.050	A/C	7.36
		S3-P30907968	3	30,907,968	4.028	T/C	7.50
		S9-P110799549	9	110,799,549	4.354	A/G	7.39
		S10-P87179990	10	87,179,990	4.201	C/T	7.61
		S11-P12773211	11	12,773,211	4.033	T/A	6.80
		S13-P6815607	13	6,815,607	4.256	G/A	7.77
		S14-P34150418	14	34,150,418	4.272	A/T	7.50
		S14-P107071402	14	107,071,402	4.282	G/A	7.42
		SCONTIG736-P863256	CONTIG736	863,256	4.616	G/A	8.55

**Table 7:** SNP loci were significantly associated with mature leaf size (MLZ) using cMLM-P + K in the three years

SNPs	Chromosome	Position	− Log_10_^P^	R^2^(%)	Allele	Year		
						2019	2020	2021
S1-P96765712	1	96,765,712	4.49	7.85	A/G	√		
S1-P122201532	1	122,201,532	5.30	9.59	T/G	√	√	√
			4.54	8.30				
			5.25	9.74				
S3-P116917717	3	116,917,717	4.62	8.75	C/T	√		
S3-P69973921	3	69,973,921	4.00	9.00	C/T		√	
S6-P124718592	6	124,718,592	4.08	7.27	G/A	√		√
			4.16	7.74				
S7-P15417610	7	15,417,610	4.02	7.86	G/A	√		
S8_P52030704	8	52,030,704	4.37	8.67	G/A	√		
S8_P52030778	8	52,030,778	4.16	8.47	G/A	√		
S9-P27970985	9	27,970,985	4.38	8.62	C/T	√		√
			4.75	8.36				
S9-P163812618	9	163,812,618	4.78	8.82	A/G	√	√	
			4.70	8.77				
			4.23	7.97				
S9-P27970980	9	27,970,980	4.02	7.79	G/C		√	√
			4.07	7.89				
S10-P78788354	10	78,788,354	4.28	8.61	G/A	√		
S11-P100806529	11	100,806,529	4.10	8.64	G/A		√	
S12-P11244003	12	11,244,003	4.16	7.72	C/T	√		
S12-P38652091	12	38,652,091	4.94	9.50	T/C	√		
S13-P92754559	13	92,754,559	4.02	6.85	A/C	√		
S13-P110363923	13	110,363,923	4.11	7.58	T/C	√		
S13-P110363973	13	110,363,973	4.04	7.46	G/A	√		
S14_P31141612	14	31,141,612	4.36	8.82	G/A	√		
S14_P31141847	14	31,141,847	4.21	8.75	G/C	√		
SCONTIG419-P527748	CONTIG419	527,748	4.48	9.33	C/T	√	√	
			4.38	9.79				
SCONTIG758-P387428	CONTIG758	387,428	4.12	7.68	A/T	√

**Table 8:** Functional annotation of candidate genes significantly associated with four leaf traits

Traits	SNP locus	Chromosome	Candidate gene	Function annotation
MLZ	S1_P122201532	1	TEA005350.1	Tetratripeptide repeat
	S9_P163812618	9	TEA029641.1	PFP-α: pyrophosphate fructose 6-phosphate 1-phosphate transferase subunit α
MLC	S1_P1076408	1	TEA027527.1	RAB geranyl geranyl transferase type 2 subunit β1
MLT	S8_P129108645	8	TEA021901.1	1,3-beta-glucan synthase
	S11_P101072100	11	TEA002469.1	Beta-glucosidase 12
MLS	S2_P130091909	2	TEA026128.1	Protein containing lob (lateral organ boundary) domain 22

### RT-qPCR analysis

**Fig. 6:**
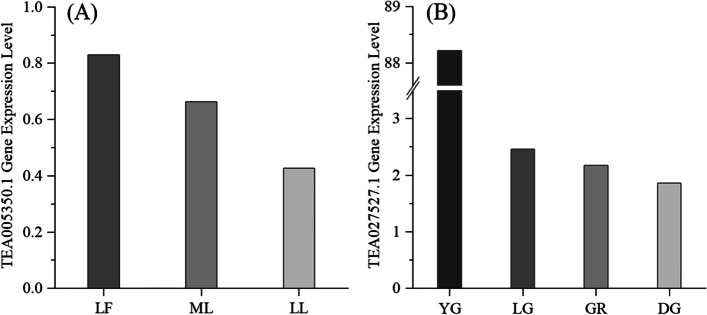
**A** The expression level of TEA005350.1 gene, a candidate gene associated with mature leaf size (MLZ), in leaflet, middle, and large leaves. L.F.: leaflet; M.L.: middle leaf; L.L.: large leaf. **B** Expression level of TEA027527.1 gene associated with mature leaf color (MLC) in yellow-green, light green, green, and dark green leaves. Y.G.: yellow-green; L.G.: light green; G.R.: green; D.G.: dark green

To determine whether potential candidate genes were involved in the accumulation of leaf size and leaf color, we determined the expression level of the TEA005350.1 gene in three leaf sizes (leaflet, middle leaf, large leaf) and TEA027527.1 gene in four leaf colors (yellow-green, light green, green, dark green) by RT-qPCR. The results showed that the TEA005350.1 gene was differentially expressed in tea plant leaflets, middle leaves, and large leaves. The expression level was highest in leaflets (Fig. 6A, Table S7). Therefore, the TEA005350.1 gene negatively regulates the formation of tea plant leaf size. The expression level of the TEA027527.1 gene in yellow-green leaves was the highest (Fig. 6B, Table S8).

### Developing and verifying the dCAPS marker

**Fig. 7:**
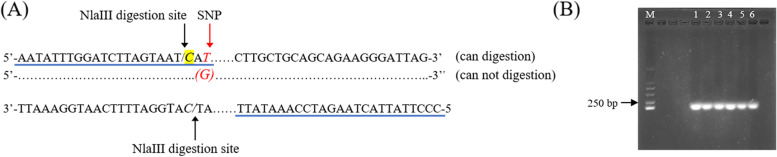
Design and verification of the dCAPS marker. **A** Establishment of a dCAPS marker with the NlaIII restriction enzyme (the underlined part is the primer sequence, italics are digestion sites, the yellow bases are the introduced one mismatch into the forward primer, the red base is the SNP site); **B** PCR products of six tea accessions using the dCAPS primer

Since an SNP mutation Chr 1 122,201,532 (T/G) in the coding region of TEA005350.1 was detected as significantly associated with MLZ, we developed a PCR-based dCAPS marker that can be used in molecular marker-assisted breeding. In this study, we introduced a “one base” mismatch in the forward primer to design dCAPS markers and developed a pair of dCAPS marker primers using restriction endonuclease Nlalll, whose digestion site was CATG (Fig. 7A). To verify the reliability of dCAPS markers, we applied the primers to six tea accessions with known MLZ genotypes, and all obtained 216 bp of PCR products (Fig. 7B, Figure S3), which indicates that the dCAPS markers developed can be used for PCR amplification.

## Discussion

The plant materials used for the association study should have a wide range of genetic diversity to capture historical recombination events in maximum quantity, so it was extremely important to select plant materials. A core collection is considered a small set representing the maximum diversity of the raw accession collection [33]. Although commercial varieties of tea, including “Fuding Dabaicha” and “Longjing”, have been widely planted in southwestern China due to their high yield and absolute economic values, many wild tea germplasm resources in Guizhou province have not been exploited. Guizhou, one of the original centers of the tea plants, is rich in wild tea tree germplasm resources. Tea is the most drank beverage in the world, processed through the young leaves. Leaf-related traits affect plant architecture, yield, and quality potential in tea plants. In this study, the investigation of four leaf traits in 338 individuals indicated that four leaf traits of 338 tea plants displayed broad variation. Moreover, a total of 100,829 high-quality SNPs were obtained from 338 tea accessions, and the number of SNPs was higher than that the previous study reported of 415 Guizhou tea accessions with 79,016 high-quality SNPs [4], suggesting that 338 tea accessions of Guizhou province are rich in genetic diversity and can be used in GWAS [4].

Based on the population structural analysis, the 338 tea plants were classified into five groups (four ancestral groups (G-I, G-II, G-III and G-IV) and one admixture group (G-V)), consistent with the phylogenetic analysis and PCA results. G–IV represented ancient landraces, including *C. sinensis* (95.57%) and *C. remotiserrata* (4.43%). A reasonable explanation about this result may be that the ancient landraces grew on the edge of terraced fields or served as fences to separate fields owned by different farmers [4]. Due to human activities, the geographic isolation between the ancient landraces and *C. remotiserrata* (wild tree) was narrowed, thus promoting cross-pollination between different germplasm, and the *C. remotiserrata* gradually acquired the genetic background of the landraces [4]. However, previous studies had revealed that 253 tea accessions from Guizhou and 100 tea accessions from Hunan were classified into three groups, which were significantly different from our 338 individuals divided into five groups due to the rich genetic diversity of C. *remotiserrata* and C. *tachangensis* tea population. Moreover, our results further showed that tea plants from the Zunyi, Bijie, and Southwest Guizhou Autonomous Prefectures were a sister branch to the cultivars, which is consistent with the results of a previous study [4].

GWASs are considered a powerful strategy for exploring the genetic basis of complex traits at the genome-wide level [34–37]. GBS-based GWAS analysis has been widely used in many horticultural plants and crops, such as tea plants, peaches, octoploid strawberry plants, melons, and radishes. For example, the GBS method was used to detect the 18,373 SNPs of 220 Brazilian peach germplasms, and the result of GWAS revealed that 13 SNPs were associated with five peach fruit traits [38]. Kishor et al. [39] revealed that 18 significant SNPs from 48 commercial *Oriental melon* varieties were associated with various morphological traits, and four potential candidate genes were annotated. Lee et al. [40] investigated the genetic diversity of 225 radish accessions and identified 44 SNPs and 20 potential candidate genes significantly associated with Fusarium wilt resistance. Compared with other crops, there are few studies based on GBS to perform GWAS of leaf traits in tea plants. Lu et al. [7] reported that eight high-quality SNPs corresponding to six candidate genes were significantly associated with three leaf traits such as leaf color, depth of leaf serration, and density of leaf serration by using a total of 8,082,370 high-quality SNPs identified from 120 ancient tea plants. In our previous report [6], nine SNPs were found to be significantly associated with four leaf traits (MLA, MLL, MLW, and MLS) using GBS-based GWAS, but no corresponding candidate gene was found.

Although we used the same method for phenotype analysis of MLZ, MLC, MLS, and MLT for three consecutive years, the phenotypes of MLC, MLS, and MLT were consistent over 3 years, and only the phenotype of MLZ still showed some changes, resulting in some differences in the identified SNPs associated with MLZ between the three years. This is mainly related to the fact that the environment greatly affects the quantitative trait MLZ of leaves [6]. To reduce the environmental impacts, we used three years of the data of MLZ for comprehensive analysis in this study.

GLM (GLM-Q, GLM-P), MLM (MLM-P + K, MLM-Q + K), and cMLM (cMLM-P + K, cMLM-Q + K) were the six most common statistical models in GWAS. An advantage of GLM is that it has a wide detection range and can detect many SNPs associated with the target traits, but its detection accuracy is not as accurate as MLM. Although MLM can improve the accuracy of GWAS by taking the kinship matrix as a random effect, it may also filter out some SNPs markers that are truly related to the target traits due to its strict control standard. The cMLM model aims to redetect those false negative SNPs filtered out by the MLM model [2]. Based on the characteristics of these six models, we selected the most appropriate model for each trait in this study for GWAS. In this study, a total of 59 high-quality SNPs were significantly associated with four leaf traits, such as MLC (nine SNPs) by using the GLM-P model, MLT (18 SNPs) by using GLM-Q model, MLZ (22 SNPs) by using cMLM-P + k model, and MLS (10 SNPs) by using the cMLM-P + k model.

LD decay distance was used to determine the region of potential candidate genes in GWAS. In Xu’s study, an SNP located on chromosome 19 associated with the starch content of leaf in Nicotiana tabacum was selected for linkage disequilibrium analysis to obtain the target candidate region [41]. Compared with self-pollinated species, the LD of cross-pollinated species, such as tea plants, declines faster because the recombination efficiency of the latter was lower [42, 43]. Our results showed that the LD levelness was the highest at *r*^2^ = 0.24, gradually beginning to decrease at *r*^2^ = 0.12 when the physical distance increased to 7 kb, and low (*r*^2^ = 0.035) when the physical distance increased to 50 kb. The result was lower than self-pollination plants *Prunus avium* [44] and rice [20]. This may be due to the self-incompatibility of tea plants [45]. Based on LD decay distance, genes located in the 7 kb region around SNPs associated with the four leaf traits were identified as likely candidate genes. However, based on the current reference genome, no functionally annotated genes were found in this region. We selected 50 kb as the reasonable distance that caused LD between genes and traits associated with SNPs because we found that LD became too low when the physical distance increased by more than 50 kb. Therefore, we broadened this region to 50 kb [2] and found 26 candidate genes, which methods had been adopted in many other GWAS cases [46, 47].

Further analysis revealed that one SNP (P-1076408) associated with MLC traits was distributed in coding sequences of the TEA027527.1 gene. Three SNPs (P-129108645, P-129108677, and P-101072100) were associated with MLT. The corresponding two functional genes were TEA021901.1 and TEA002469.1, respectively, and only one SNP (P-130091909) distributed in the coding sequence of the TEA026128.1 gene was associated with MLS. Moreover, two SNPs (P-122201532, P-163812618) distributed in the coding sequences of the TEA005350.1 gene and TEA029641.1 gene, respectively, were associated with MLZ within the three years. In addition, the functions of these candidate genes were further investigated based on the homologous genes in *Arabidopsis* and KEGG analysis [48–50], which were verified by RT-qPCR.

The shape of leaves is determined by development processes [51]. At first, the leaf is a small protuberance on the periphery of the shoot tip meristem (SAM), then undergoes asymmetric growth, expansion and maturation, and finally forms a shape [52]. The TEA026128.1 gene belongs to the plant-specific LBD (Lateral Organ Boundaries Domain) gene family. It is expressed in the proximal base of the initial lateral organ and is crucial in regulating plant lateral organ development. We searched the TEA026128.1 gene using the TPIA database and found it was annotated as LOB domain-containing protein 22 (*CsLBD22*). By constructing a phylogenetic tree, Teng et al. [53] have proved that the *CsLBD22* gene of the tea plant and the *AtLBD15* gene of *Arabidopsis thaliana* are orthologous genes. There is evidence that the *AtLBD15* gene is involved in the development of shoot tip meristem and regulates the expression of WUSCHEL [54]. In addition, Shuai et al. [55] showed that the lateral organ boundary (LOB) family genes were expressed in the adaxial side of the lateral organ base, and their ectopic expression led to changes in leaf shape. Our study found that the TEA026128.1 gene (*CsLBD22*) was located within 9.40 kb downstream of SNP significantly associated with MLS traits, proving that the TEA026128.1 gene is a potential candidate gene involved in the formation of MLS in tea plants.

Cell proliferation and expansion can change the number and size of cells during leaf growth, thus affecting the final leaf size [56]. However, cell proliferation and expansion are closely related to cell cycle regulation [56]. The TEA005350.1 gene belongs to the tetratricopeptide-like helix domain superfamily; it encodes a protein that plays an important role in cell division control and plant morphogenesis, mediates protein interactions, and participates in cell cycle regulation. Our results show that the *AtTSK* gene of Arabidopsis had high homology with tea plant TEA005350.1 by Blasting in the TPIA database. Therefore, we annotated the TEA005350.1 gene regarding the function of the *AtTSK* gene. We determined the expression level of the TEA005350.1 gene in three leaf sizes (leaflet, middle leaf, large leaf) by RT-qPCR. The results showed that the TEA005350.1 gene was differentially expressed in tea plant leaflet, middle leaf, and large leaves. The expression level was highest in leaflets. There is evidence that the *AtTSK* gene is expressed in the S phase of the cell cycle, and its defect delays the G2/M transformation process of the Arabidopsis cell cycle, resulting in the accumulation of many cells in the G2 phase [57]. The *AtTSK* gene was also involved in regulating cell division of the shoot tip meristem of *A. thaliana*, and its mutant meristem cells were larger than the wild type [58]. Protein TONSOKU has been studied in *A. thaliana*, *Nicotiana attenuata*, *Helianthus annuus*, and *Dendrobium catenatum*, but there is no relevant report on tea plants at present [59–61], which further confirmed our findings that the TEA005350.1 gene is located 36.84 kb downstream of the SNP (on chromosome 1) significantly associated with MLZ traits is a potential candidate gene associated with MLZ traits of tea plants. Therefore, verifying the accuracy of this gene associated with the mature leaf size of tea plants will be part of our subsequent work.

The TEA029641.1 gene belongs to the Phosphofructokinase superfamily and can catalyze the reversible mutual transformation of fructose-6-phosphate and fructose-1,6-diphosphate, which is a key regulatory step in the glycolysis pathway [62]. Fructose pyrophosphate 6 phosphate 1 phosphotransferase subunit α also participates in the pentose phosphate pathway, fructose and mannose metabolism, and other biological pathways. In RNAi transgenic lines, the homologous gene *AtPFP-α* of TEA029641.1 in *A. thaliana*, showed significant growth retardation and smaller rosette leaves [63], indicating that the *AtPFP-α* gene affects the size of rosette leaf of Arabidopsis. In plants, glycolysis is the main respiration route and provides ATP, reductant, and precursor materials required for plant growth and development [64]. Therefore, we speculate that the TEA029641.1 gene, which is located within 1 kb downstream of SNP, was significantly associated with MLZ and was a candidate gene of MLZ.

The color of leaves was affected by the kind, content, and proportion of pigments in leaves, among which chlorophyll, carotenoids, and anthocyanins were the most important pigments [65]. The TEA027527.1 gene was annotated as geranylgeranyl transfer type-2 subunit beta-like, referring to TPIA and NCBI databases. It belonged to the protein isoprene transferases family and could catalyze geranylgeranyl pyrophosphate synthase, playing an important role in protein isoprene modification [66]. There is evidence that the rate and yield of carotenoid synthesis from geranylgeranyl pyrophosphate (*GGPP*) were guided by geranylgeranyl pyrophosphate synthase (*GGPPS*) [67]. Based on the TPIA database, the *RGTB1* gene in *A. thaliana* was a homologous gene of the TEA027527.1 gene in the tea plant, and its mutant has defects in etiolation. It has been reported that specific RAB GTPase is up-regulated in etiolated wild-type plants [68]. This found that the TEA027527.1 gene was located at 25.57 kb upstream of SNP on chromosome 1 and was significantly associated with MLC traits. Therefore, the TEA027527.1 gene may be a candidate gene for MLC.

Texture characteristics are closely related to the composition (pectin, cellulose, hemicellulose) of cell walls [69], the destruction of which will make cells lose their support and become soft [70]. Based on the annotation information of the TPIA database, we found that the TEA021901.1 gene and TEA002469.1 gene were annotated as callose synthase 11-like and belonging to the Glycosyl transfer family, and beta-glucosidase 12-like belonged to the Glycoside hydrolase family (GH1). It has been reported that callose is produced by callose synthase, which is one of the basic structures of the cell wall [71]. The GH1 family has glycoside hydrolase activity and can hydrolyze β-D-galactoside and L-arabinoside. Studies by Guo et al. [72] and Yang et al. [69] showed that (1 → 4)-β-Galactose and (1 → 5)-α-Arabinose are the main components of the cell wall pectin side chain, and the content of pectin was positively correlated with the hardness. Therefore, the TEA002469.1 gene located at 0.03 kb upstream of SNP on chromosome 8 and the TEA002469.1 gene located at 26.9 kb upstream of SNP on chromosome 11 may be candidate genes associated with MLT traits.

Genome-wide association analysis has identified many SNP associated with leaf morphological characteristics [73, 74]. However, marker-assisted selection (MAS) breeding is still relatively less applied. One of the main reasons for this is the lack of effective markers. For breeders, these markers have a high reference value for identifying traits of interest. Wang et al. [2] introduced two base mismatching restriction endonuclease EcoR1 and developed a TBF dCAPS marker for MAS breeding of *C.sinensis*. Hu et al. [56] developed a dCAPS polymorphic marker related to melon seed coat color to construct a genetic map after genotyping the population with insertion-deletion (InDels). In this study, we introduced a base mismatch to develop a dCAPS marker associated with MLZ and verified its feasibility in materials. The development of this dCAPS marker will provide a reference for screening molecular markers closely related to leaf size phenotypic traits.

## Conclusions

In this study, we applied GBS to the GWAS of 338 tea accessions genotypes and MLZ, MLC, MLT, and MLS. We found 22, 9, 18, and ten SNPs associated with MLZ, MLC, MLT, and MLS, respectively, in three years. Using common recognition in the same model (cMLM-P + K) of MLZ within the three years, a final SNP (P1-122,201,532) was obtained, proving that it has a relatively strong correlation with MLZ. Then, we searched for candidate genes in the 50 kb region upstream and downstream of the final important SNP site and found two (TEA005350.1, TEA029641.1), one (TEA027527.1), two (TEA021901.1, TEA002469.1), and one (TEA026128.1) potential candidate genes associated with MLZ, MLC, MLT, and MLS, respectively. Based on the strong correlation with MLZ, the expression level of the TEA005350.1 gene in three different types of mature leaves was detected by RT-qPCR. At present, there are few reports on the TEA027527.1 gene associated with MLC. We used RT-qPCR to detect its expression level in different types of mature leaf colors. The TEA005350.1 and TEA027527.1 genes showed differential expression levels. In addition, a dCAPS marker associated with MLZ was designed, which may be helpful for future MAS breeding. Our research demonstrates that GBS-based GWAS is an effective method for analyzing complex traits and finding candidate genes in tea plants.

## Methods

### Plant materials and phenotyping

**Fig. 8:**
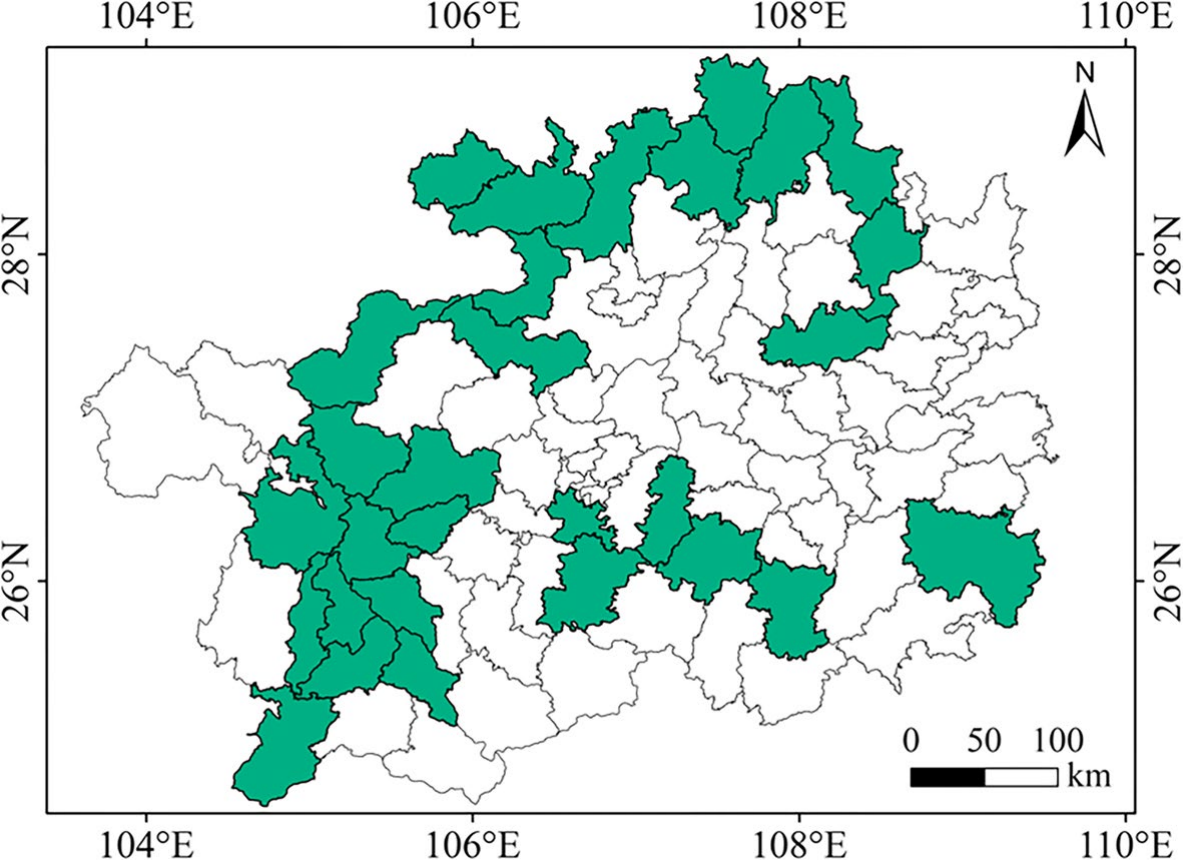
Distribution of 336 materials from 30 counties in Guizhou Province

**Fig. 9:**
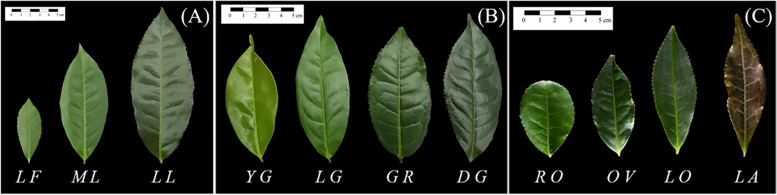
**A** Three morphological characteristics of “Mature leaf size (MLZ)” phenotypic trait. L.F.: leaflet; M.L.: middle leaf; L.L.: large leaf. **B** Morphological characteristics of four “Mature leaf colors (MLC)”. Y.G.: yellow-green; L.G.: light green; G.R.: green; D.G.: dark green. **C** Morphological characteristics of four “Mature leaf shapes (MLS)”. R.O.: round; O.V.: oval; L.O.: long oval; L.A.: lanceolate

A total of 338 tea plant accessions, including 202 cultivated tea plants and 136 wild-type tea plants, were used for this study (Table S5). Among them, 336 accessions were collected from 30 counties in Guizhou Province (Fig. 8), and the other two varieties were collected from Fujian and Zhejiang Province, respectively (Table S5). The materials were planted in the tea genetic resource nursery (106° 40′ E and 26° 20′ N) at Guizhou University and were collected for GBS sequencing. We further investigated four leaf traits of tea plants: mature leaf size (MLZ: Leaflet area < 20 cm^2^, 20 cm^2^ < Middle leaf area < 40 cm^2^, 40 cm^2^ < Large leaf area < 60 cm^2^) (Fig. 9A); mature leaf color (MLC: Yellow-green, Light green, Green, and Dark green) (Fig. 9B); mature leaf shape (MLS: Oval, Long oval, Lanceolate, Round) (Fig. 9C); and mature leaf texture (MLT: Soft, Medium, Hard). In 2019, 2020, and 2021 (these three consecutive years were regarded as three independent environments), a field experiment was conducted to investigate the above four traits on five mature leaves of 338 tea accessions according to descriptors for tea germplasm resources (NY/T 2943–2016) and assign values to qualitative traits by the scoring method [30] (Table S9). For quantitative trait MLZ, the average phenotypic value of each year in three years was considered for association analysis. Qualitative traits (MLC, MLT, MLS) were verified in the second and third-year surveys, and no significant difference was observed between the three years’ phenotypic values; we used one year’s data for association analysis (Table S9). The descriptive statistical analysis of four phenotypic traits (e.g., mean, minimum and maximum, standard deviation (SD), and coefficient of variation (CV)) was used to evaluate the population diversity of 338 tea accessions. SPSS estimated analysis of variance (ANOVA) of MLZ based on the average value of each character added in the three years. The component of variation assessed generalized heritability (h_B_^2^).

### DNA extraction, library construction, and sequencing

A Plant Genomic DNA Rapid Extraction Kit (Beijing Biomed Gene T echnology Co. Ltd., Beijing, China) was used to extract genomic DNA. Restriction endonuclease SacI and MseI (5 U; New England Biolabs (NEB), Ipswich, USA) were used for digesting the DNA isolated from each sample. After digestion, adaptors "SacAD and MseAD" with unique barcodes were ligated to the digested DNA fragments. Then, the PCR Primer Cocktail and PCR Master Mix were used to amplify the purified DNA fragments. The 500–550 bp amplified fragment was retrieved through electrophoresis using 2% agarose gel, and purified using QIAquick Gel Extraction Kit (Qiagen) [75]. The average length of DNA fragments was determined using the Agilent DNA 12,000 kit and 2100 Bioanalyzer system (Agilent). Quantitative real-time PCR with a TaqMan probe was used to quantify the final DNA library and the Illumina HiSeq X ten platform with the paired-end 150 (PE150) sequencing strategy was used to sequence [75].

### Sequence alignment and SNP identification

We used barcodes to de-multiplex the raw DNA reads and trim the adaptors with a custom perl script. Ultimately, the reads with quality values > 5 were retained as the clean data, then the BWA-MEM v.0.7.10 with parameters ‘-T 20 -k 30’ was used to align with the reference genome (Accession Number. SRA536878, http://tpia.teaplant.org/) [76, 77]. GATK v.3.7.0 (https://github.com/broadinstitute/gatk/releases) was used call for SNPs [78]. Based on the method of Chen et al. [79], Hussain et al. [80], and Eltaher et al. [81], we summarized and then used the following filtering criteria: (1) The variants were bi-allelic SNPs; (2) “QUAL < 50.0 || QD < 2.0 || FS > 60.0 || MQ < 40.0 || Mapping Quality Rank Sum < -12.5 || Read Pos Rank Sum < -8.0” were used in GATK v. 3.7.0 (https://github.com/broadinstitute/gatk/releases) to filter the SNPs; (3) Minor allele frequency (MAF) lower than 0.05 or missing data rate higher than 20% were filtered out by VCFtools v.0.1.16 (https://github.com/vcftoools/vcftools) [82]. Three-hundred-thirty-eight accessions and 100,829 SNPs were retained for further analysis after the filter. Genetic diversity analysis.

Plink v.1.90 (https://www.cog-genomics.org/plink2/) [83] was used to calculate *Ho*, *He*, *MAF*, and *Fis* for each inferred population. VCFtools was used to calculate *Pi* and *Tajima’s D* for each inferred population [82]. SPSS v.25 (IBM Corp., Armonk, NY, USA) was used to determine significant differences between these indices [84].

### Population structure, PCA, and phylogenetic analysis

Admixture v.1.3.0 [85] was used to estimate the proportions of admixtures among the 338 tea accession populations by running k = 2–9. Then, the best value of K was determined by cross-validation (CV) and log-likelihood estimates. We set the threshold to 0.8 to distinguish the pure group from the mixed group. PCA was analyzed by Tassel v.5.2.43 [86]. The neighbor-joining tree was constructed using MEGA-X [87].

### Linkage disequilibrium analysis

Based on the allele frequency correlation (r^2^), the PopLDdecay V.3.30 was used to calculate the paired linkage disequilibrium of 29,393,327 genome-wide unfiltered SNPs from more than 500 kb, and the LD attenuation map was generated [88].

### Genome-wide association study

To ensure the accuracy of the results, SNPs with a minor allele frequency less than 0.05 or a maximum deletion genotype frequency greater than 20% were screened for GWAS. The six linear regression models were the general linear model (GLM) (GLM-Q and GLM-P), mixed linear model (MLM) (MLM-Q + K and MLM-P + K), and compressed mixed linear model (cMLM) (cMLM-Q + K and cMLM-P + K). The Q-matrix (Q) or PCA-matrix (P) was taken as the fixed effect in GLM models to control possible false positives resulting from the confounding of population structure. The kinship matrix (K) was considered a covariate factor in MLMs and cMLMs to reduce unequal relatedness among genotypes [89]. K was the kinship matrix constructed by the TASSEL software, the best admission results representing population membership were denoted as Q, and P was the result of principal component analysis.. The Quantile–Quantile plots (Q-Q plots) showed the fit between the model and the data, which was used to judge the deviation between the observed and expected values. Manhattan plots showed the correlation between SNPs sites on each chromosome and this trait. Comparing the Q-Q plots of the output of the six GWAS models, the best model fitting the curve of the expected value to the observed value was entered into the later analysis as the optimal model for each trait [87]. -Log_10_^(P)^ ≥ 4.0 [90] was selected as the threshold to identify SNPs sites closely associated with four leaf traits.

### Candidate genes prediction

We used the best model of four traits to screen significant SNPs to reduce false positives, and candidate genes were searched within 50 kb upstream and downstream of the linked SNP loci. The functional annotation information of candidate genes was obtained from National Center for Biotechnology Information Database (NCBI, https://www.ncbi.nlm.nih.gov/) and the candidate genes that may be related to traits were predicted combined with Tea Plant Information Archive Database (TPIA, http://tpia.teaplant.org/).

### Extraction of RNA and RT-qPCR analysis

We selected 15 tea materials (including 5 large leaf materials, 5 middle leaf materials and 5 leaflet materials) with different leaf sizes and 20 tea materials with different leaf colors (including 5 yellow green materials, 5 light green materials, 5 green samples and 5 dark green materials) for RT-qPCR. For reverse transcription, the total RNA of 35 tea accessions was extracted using the UNIQ-10 column Trizol total RNA Extraction Kit (Sangon Biotech Co. Ltd., Shanghai). RT-qPCR was performed using ChamQ Universal SYBR qPCR master Mix Kit (novozan Biology), using cDNA as a template to detect the expression level of the TEA005350.1 and TEA027527.1 genes in tea varieties with different leaf sizes and colors. The results were analyzed using the 2^ ^(−△△Ct)^ [91] method, and GADPH (Forward primer: AGCTGCACAACCAACTGTTTG, Reverse primer: AGCTGCACAACCAACTGTTTG) was used as an internal reference gene for relative quantification analysis with three replicates for each sample.

### Developing and verifying a dCAPS marker associated with MLZ

SnpEff software was used to annotate the.vcf sequence files for obtaining SNP mutation site information, and we used the TPIA database to call the genes’ coding sequences (CDSs). In addition, the online software dCAPS Finder 2.0 (http://helix.wustl.edu/dcaps/dcaps.html) was used to design markers at about 200 kb upstream and downstream of the SNP site and found the corresponding specific endonuclease digestion sites. Primer Premier 5.0 software was used to design specific reverse primers. Finally, genomic DNA was extracted from six tea accessions with known MLZ, and the designed markers were used for PCR amplification to verify the feasibility of the dCAPS markers obtained.

## Supplementary Information

**Additional file 1: Figure S1.** Dynamics of population structure under different K (K = 2-9) values of 338 tea accessions. **Figure S2.** The Q-Q plots and Manhattan plots of other models of 4 traits except the optimal model. (A1) MLZ-2019-cMLM-Q+K; (A2) MLZ-2019-GLM-P; (A3) MLZ-2019-GLM-Q; (A4) MLZ-2019-MLM-P+K; (A5) MLZ-2019-MLM-Q+K; (B1) MLZ-2020-cMLM-Q+K; (B2) MLZ- 2020-GLM-P; (B3) MLZ-2020-GLM-Q; (B4) MLZ-2020-MLM-P+K; (B5) MLZ-2020-MLM-Q+K; (C1) MLZ-2021-cMLM-Q+K; (C2) MLZ-2021-GLM-P; (C3) MLZ-2021-GLM-Q; (C4) MLZ-2021-MLM-P+K; (C5) MLZ-2021-MLM-Q+K; (D1) MLC-cMLM-P+K; (D2) MLC-cMLM-Q+K; (D3) MLC-GLM-P; (D4) MLC-MLM-P+K; (D5) MLC-MLM-Q+K; (E1) MLS-cMLM-Q+K; (E2) MLS-GLM-P; (E3) MLS-GLM-Q; (E4) MLS-MLM-P+K; (E5) MLS-MLM-Q+K; (F1) MLT-cMLM-P+K; (F2) MLT-cMLM-Q+K; (F3) MLT-GLM-Q; (F4) MLT-MLM-P+K; (F5) MLT-MLM-Q+K. **Figure S3.** The labeled complete gels of PCR products of six tea accessions using the dCAPS primer. **Table S1.** The quality control (QC) data of each sample. **Table S2.** Genotyping of 100,829 SNPs based on GBS in 168 tea accessions. **Table S3.** Genotyping of 100,829 SNPs based on GBS in 170 tea accessions. **Table S4.** Distribution information of 100,829 SNPs on 15 chromosomes of tea plant.**Table S5.** Information of 338 tea accessions used in the present study. **Table S6.** Statistics of the number and ratio of the accessions of species, and both cultivation status in five inferred populations.**Table S7.** Analysis of TEA005350.1 gene expression of 15 tea plant accessions. **Table S8.** Analysis of TEA027527.1 gene expression of 20 tea plant accessions. **Table S9.** Four leaf phenotypic traits (mature leaf size, mature leaf color, mature leaf shape and mature leaf texture) data and their assignment of 338 tea accessions in 2019, 2020 and 2021, respectively.

## Data Availability

The plant materials were growing in our resource nursery which are available from the corresponding author on reasonable request. The raw sequence data reported in this study have been deposited in the Genome Sequence Archive in BIG Data Center, Beijing Institute of Genomics (BIG), Chinese Academy of Sciences, under accession number CRA001438 that is publicly accessible at http://bigd.big.ac.cn/gsa.

## Abbreviations

MLZ: Mature leaf size
MLC: Mature leaf color
MLS: Mature leaf shape
MLT: Mature leaf texture
SNPs: Single nucleotide polymorphisms
CVerror: Cross-validation error
GBS: Genotyping-by-sequencing
GWAS: Genome-wide association study
LD: Linkage disequilibrium
NJ tree: Neighbor-Joining tree
PCA: Principal component analysis
RT-qPCR: Reverse transcription quantitative polymerase chain reaction
dCAPS: Derived cleaved amplified polymorphism
