# Supplementary material for: Genome-wide association study of leaf-related traits in tea plant in Guizhou based on genotyping-by-sequencing
Source: BMC Plant Biol. 2023 Apr 12;23:196. doi: 10.1186/s12870-023-04192-0 (PMC10091845; doi:10.1186/s12870-023-04192-0)
Supplement: Supplementary file 1 — Additional file 1: Figure S1. Dynamics of population structure under different K (K = 2-9) values of 338 tea accessions. Figure S2. The Q-Q plots and Manhattan plots of other models of 4 traits except the optimal model. (A1) MLZ-2019-cMLM-Q+K; (A2) MLZ-2019-GLM-P; (A3) MLZ-2019-GLM-Q; (A4) MLZ-2019-MLM-P+K; (A5) MLZ-2019-MLM-Q+K; (B1) MLZ-2020-cMLM-Q+K; (B2) MLZ- 2020-GLM-P; (B3) MLZ-2020-GLM-Q; (B4) MLZ-2020-MLM-P+K; (B5) MLZ-2020-MLM-Q+K; (C1) MLZ-2021-cMLM-Q+K; (C2) MLZ-2021-GLM-P; (C3) MLZ-2021-GLM-Q; (C4) MLZ-2021-MLM-P+K; (C5) MLZ-2021-MLM-Q+K; (D1) MLC-cMLM-P+K; (D2) MLC-cMLM-Q+K; (D3) MLC-GLM-P; (D4) MLC-MLM-P+K; (D5) MLC-MLM-Q+K; (E1) MLS-cMLM-Q+K; (E2) MLS-GLM-P; (E3) MLS-GLM-Q; (E4) MLS-MLM-P+K; (E5) MLS-MLM-Q+K; (F1) MLT-cMLM-P+K; (F2) MLT-cMLM-Q+K; (F3) MLT-GLM-Q; (F4) MLT-MLM-P+K; (F5) MLT-MLM-Q+K. Figure S3. The labeled complete gels of PCR products of six tea accessions using the dCAPS primer. Table S1. The quality control (QC) data of each sample. Table S2. Genotyping of 100,829 SNPs based on GBS in 168 tea accessions. Table S3. Genotyping of 100,829 SNPs based on GBS in 170 tea accessions. Table S4. Distribution information of 100,829 SNPs on 15 chromosomes of tea plant.Table S5. Information of 338 tea accessions used in the present study. Table S6. Statistics of the number and ratio of the accessions of species, and both cultivation status in five inferred populations.Table S7. Analysis of TEA005350.1 gene expression of 15 tea plant accessions. Table S8. Analysis of TEA027527.1 gene expression of 20 tea plant accessions. Table S9. Four leaf phenotypic traits (mature leaf size, mature leaf color, mature leaf shape and mature leaf texture) data and their assignment of 338 tea accessions in 2019, 2020 and 2021, respectively. [file 12870_2023_4192_MOESM1_ESM.zip › Figure S2.docx]

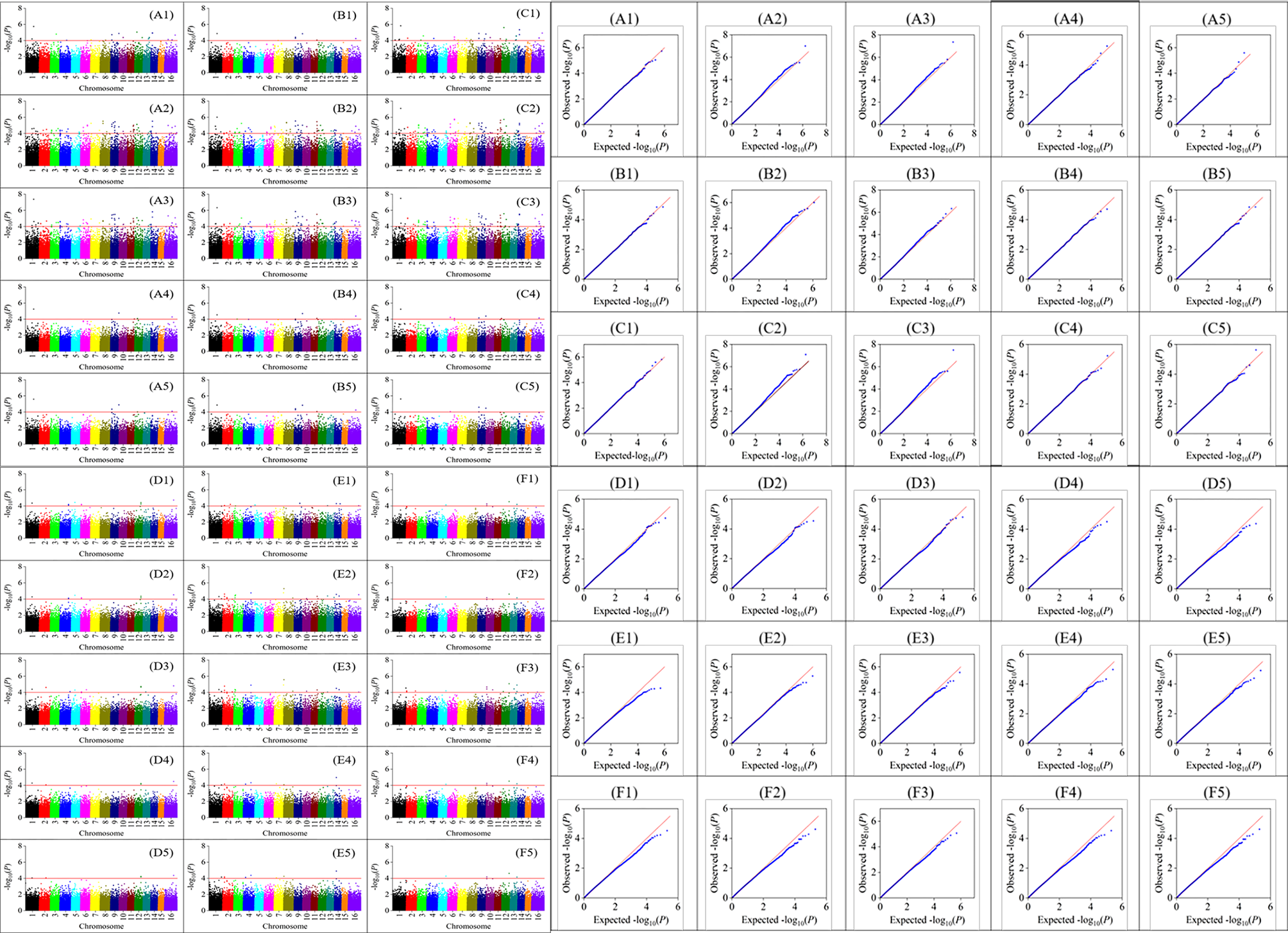


**Figure S2.** The Q-Q plots and Manhattan plots of other models of 4 traits except the optimal model. (A1) MLZ-2019-cMLM-Q+K; (A2) MLZ-2019-GLM-P; (A3) MLZ-2019-GLM-Q; (A4) MLZ-2019-MLM-P+K; (A5) MLZ-2019-MLM-Q+K; (B1) MLZ-2020-cMLM-Q+K; (B2) MLZ- 2020-GLM-P; (B3) MLZ-2020-GLM-Q; (B4) MLZ-2020-MLM-P+K; (B5) MLZ-2020-MLM-Q+K; (C1) MLZ-2021-cMLM-Q+K; (C2) MLZ-2021-GLM-P; (C3) MLZ-2021-GLM-Q; (C4) MLZ-2021-MLM-P+K; (C5) MLZ-2021-MLM-Q+K; (D1) MLC-cMLM-P+K; (D2) MLC-cMLM-Q+K; (D3) MLC-GLM-P; (D4) MLC-MLM-P+K; (D5) MLC-MLM-Q+K; (E1) MLS-cMLM-Q+K; (E2) MLS-GLM-P; (E3) MLS-GLM-Q; (E4) MLS-MLM-P+K; (E5) MLS-MLM-Q+K; (F1) MLT-cMLM-P+K; (F2) MLT-cMLM-Q+K; (F3) MLT-GLM-Q; (F4) MLT-MLM-P+K; (F5) MLT-MLM-Q+K.
